# Subtle signs of atrial cardiomyopathy and left ventricular diastolic dysfunction are associated with reduced cognitive function: results from the Hamburg City Health Study

**DOI:** 10.1007/s00392-024-02581-5

**Published:** 2024-11-27

**Authors:** Amelie H. Ohlrogge, Stephan Camen, Lina Nagel, Jan Brederecke, Märit Jensen, Ewgenia Stenmans, Daniel Engler, Christian Schulte, Jan Albrecht, Dora Csengeri, Paulus Kirchhof, Bastian Cheng, Marvin Petersen, Carola Mayer, Christin S. Börschel, Jan-Per Wenzel, Stefan Blankenberg, Simone Kühn, Götz Thomalla, Renate B. Schnabel

**Affiliations:** 1 Department of Cardiology, University Heart and Vascular Center Hamburg, University Medical Center Hamburg-Eppendorf, Martinistraße 52, 20246 Hamburg, Germany; 2 DZHK (German Center for Cardiovascular Research), Partner Site Hamburg/Kiel/Luebeck, Hamburg, Germany; 3 Department of Psychiatry, Psychotherapy and Psychosomatics, Auguste-Viktoria-Hospital, Berlin, Germany; 4 Department of Neurology, University Medical Center Hamburg-Eppendorf, Hamburg, Germany; 5 Institute of Cardiovascular Sciences, College of Medical and Dental Sciences, Medical School, University of Birmingham, Edgbaston, Birmingham, UK; 6 Department of Rhythmology, University Heart Center Luebeck, University Hospital Schleswig-Holstein, Luebeck, Germany; 7 Department of Psychiatry and Psychotherapy, Medical Center Hamburg-Eppendorf, Hamburg, Germany; 8 Center for Environmental Neuroscience, Max Planck Institute for Human Development, Berlin, Germany

**Keywords:** Atrial cardiomyopathy, Left atrial strain, Cognitive function, Cohort study, Echocardiography

## Abstract

**Background:**

Atrial fibrillation is associated with cognitive dysfunction. Atrial cardiomyopathy has been correlated with both entities. We aimed to characterize the association of echocardiographic parameters of atrial cardiomyopathy with cognitive function and cerebral changes.

**Methods:**

Participants of the population-based Hamburg City Health Study underwent in-depth transthoracic echocardiography and cognitive function testing, the Animal Naming Test (ANT), the Trail Making Test A (TMT-A) and B (TMT-B), 10-word learning test and cerebral magnetic resonance imaging.

**Results:**

After excluding individuals with stroke or depression, data from 7852 individuals were available. In multi-variable-adjusted regression analyses, the E/e’-ratio was associated with the level of impairment in the ANT (− 0.19 per one standard deviation [SD] increase, 95% confidence interval [CI] − 0.36–[− 0.01]) and the TMT-A (0.01 per one SD increase, 95% CI 0.003–0.020). Left atrial global peak strain was associated with positive performance in the TMT-A and B (-0.01 per one SD increase [95% CI − 0.02–(− 0.002)] and − 0.02 per one SD increase [95% CI − 0.03–(− 0.01)], respectively) and the immediate recall of the 10-word learning test (0.11 per one SD increase, 95% CI 0.02–0.20). The E/e’-ratio was positively associated with the total and periventricular white matter hyperintensity load in age- and sex-adjusted regression analyses though statistical significance was lost after multi-variable adjustment.

**Conclusions:**

Subclinical echocardiographic signs of atrial cardiomyopathy and left ventricular diastolic dysfunction are associated with impaired performance in cognitive tests in the population. Our data provide evidence of the clinically important cardio-cerebral axis, relating cardiac dysfunction with cognitive performance.

**Graphical abstract:**

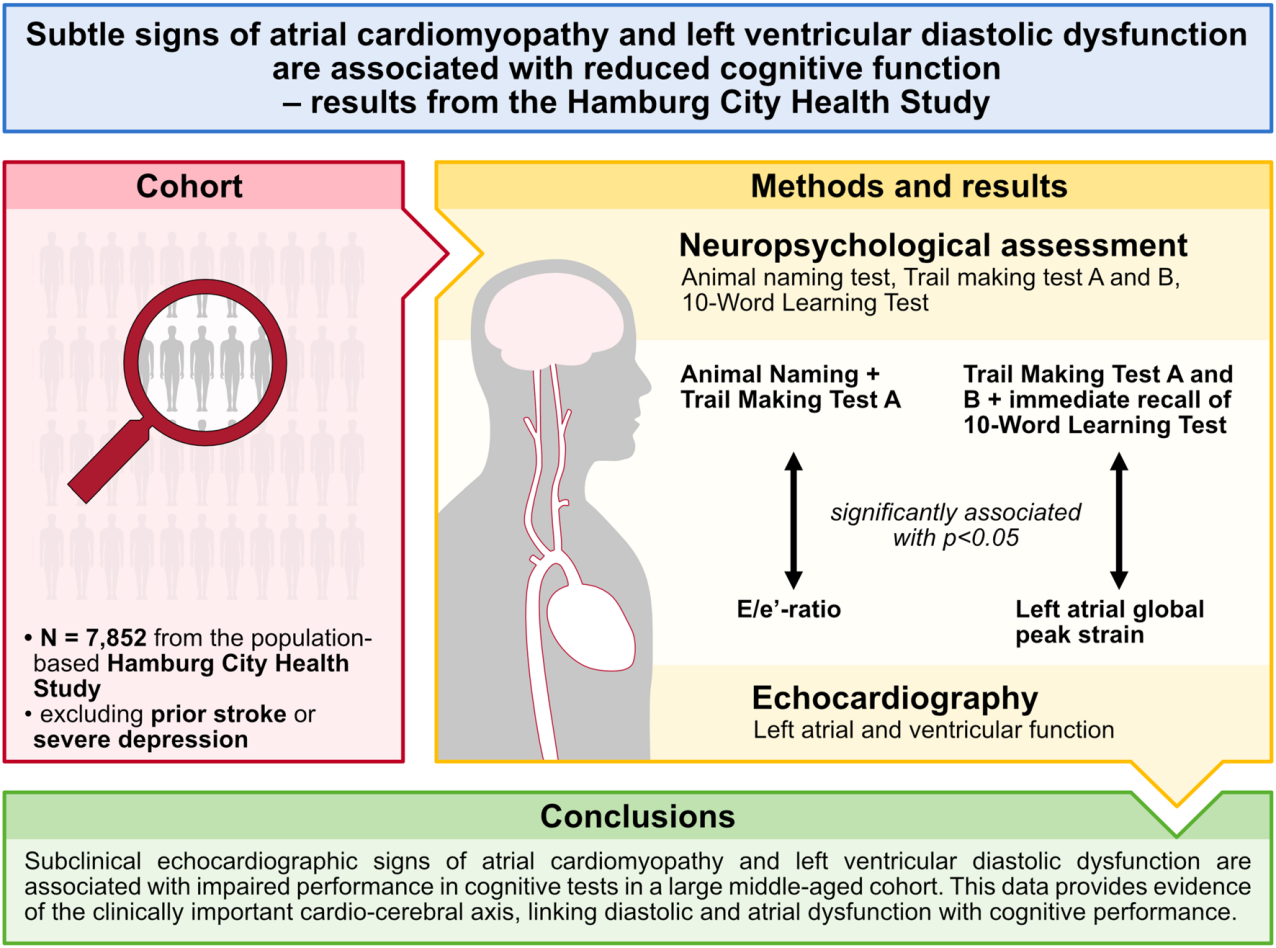

**Supplementary Information:**

The online version contains supplementary material available at 10.1007/s00392-024-02581-5.

## Introduction

Vascular cognitive impairment and dementia are caused by a variety of mechanisms, among which cortical infarcts and cerebral small vessel disease are best known [1]. Atrial fibrillation (AF) is the most common rhythm disorder and it is associated with cognitive decline and dementia [2–4]. A variety of pathological processes, including fibrosis, inflammation and.

remodeling of the atrial intercellular matrix lead to a deterioration of the atrial function. Clinically, this process can be detected by echocardiography using diastolic function and strain analyses. The resulting clinical condition of atrial cardiomyopathy strongly impacts the risk of future AF, while at the same time, there is evidence of a causal role in incident dementia [5–8]. Due to a thrombogenic milieu [7, 8], reduced atrial contractility, and potentially lower cerebral blood flow [1, 9, 10], atrial cardiomyopathy could be a precursor and a cause of cognitive dysfunction. However, data on the association of echocardiographic parameters of left ventricular (LV) diastolic function and atrial cardiomyopathy with cognitive function in individuals from the community setting are scarce. White matter hyperintensities (WMH) of presumed vascular origin are subtle signs of brain damage seen on cerebral imaging that are associated with an increased risk of cognitive decline, dementia and stroke [11, 12]. They may result from cerebral ischemia caused by cerebral small vessel disease and are linked to cardiovascular risk factors [12, 13] and AF [14, 15]. It has been hypothesized that WMH and other clinically silent brain lesions might be the structural counterpart that conveys cognitive decline in patients with AF and atrial cardiomyopathy [16]. Data on the relation of early atrial impairment and cognitive functioning and cerebral alterations in the community are rare.

The aim of the study is to investigate the association of echocardiographic parameters of LV diastolic and left atrial (LA) function and morphology with cognitive performance in individuals from the general population from the Hamburg City Health Study (HCHS). We hypothesized that LA strain, a sensitive parameter of diastolic dysfunction and atrial cardiomyopathy, will be related to subclinical impairment in cognitive function. Furthermore, we aimed to investigate associations of these echocardiographic parameters with WMH on cranial magnetic resonance imaging (cMRI).

## Methods

### Study design and participants

The HCHS enrolls inhabitants of Hamburg aged 45–74 years, identified by random sampling from the official inhabitant data file [17]. Data from the first 10,000 participants of the HCHS were available and selected for analysis. Of these, 8211 individuals received a transthoracic echocardiography examination. After exclusion of 359 individuals with prior/known stroke (*N* = 243) or severe depression (*N* = 116; defined as a score ≥ 15 in the Patient Health Questionnaire [18]), 7852 individuals were included in the final analyses for the present study.

Physical examinations were conducted by trained and certified medical staff according to standardized operation procedures and the data integrity was controlled by a detailed, predefined quality control algorithm [17]. Only quality-controlled data were used for statistical analyses in this study. A subgroup of 2069 participants with an increased risk for cerebrovascular diseases were invited to a cMRI examination.

The research protocol was approved by the local ethics committee of the Hamburg Chamber of Medical Practitioners (PV5131) as well as the Data Protection Commissioner of the University Medical Center of the University Hamburg-Eppendorf and the Data Protection Commissioner of the Free and Hanseatic City of Hamburg. The study has been registered at ClinicalTrial.gov (NCT03934957). All participants provided written informed consent.

### Definition of clinical covariates

Arterial hypertension was defined as systolic or diastolic blood pressure > 140 mmHg or > 90 mmHg, respectively, prescription of antihypertensive drugs or self-reported hypertension. Coding of diabetes mellitus was based on self-report, or a fasting plasma glucose ≥ 126 mg/dl or a non-fasting plasma glucose ≥ 200 mg/dl. Educational level was assessed according to the International Standard Classification of Education (ISCED) and divided into three groups: low (ISCED levels 0–2), medium (ISCED levels 3–4) and high (ISCED levels 5–8).

### Transthoracic echocardiography examination acquisition and analysis

Single timepoint transthoracic echocardiography was systematically performed within the baseline examination program. The patients were examined in left lateral position with the Siemens Acuson SC2000 Prime ultrasound device (Siemens Healthineers, Erlangen, Germany). The examination followed a strictly standardized protocol, which included all established standard echocardiography views as well as Doppler velocimetry. Recordings were electrocardiogram-triggered and preferably performed with breath holding technique. For image analysis and quality assurance, standardized operating procedures were defined in agreement with the current guidelines of the American Society of Echocardiography and the European Association of Cardiovascular Imaging [19, 20]. Left ventricular ejection fraction (LVEF) was calculated from LV end-diastolic and end-systolic volumes using the modified Simpson’s rule [19]. The end-diastolic thickness of the interventricular septum was assessed in the parasternal long-axis view. Maximum and minimum LA volumes, and the LA ejection fraction ($\frac{{\text{maximu}}\text{m LA volume - minimum LA volume}}{\text{maximum LA volume}}$ × 100) were calculated using the disk summation technique based on biplane measurements (apical four- and two-chamber view) [19]. If the LA volumes were only available from one plane, these values were used for analyses to avoid missing data. All volume-based measurements were indexed to body surface area.

The peak E-wave and peak A-wave velocities were recorded using pulsed wave Doppler with the sample volume placed between the mitral leaflet tips in the apical four-chamber view [20]. The peak e’-velocities were assessed using tissue Doppler imaging by placing the pulsed Doppler sample volume at the lateral (lateral e’) and septal (septal e’) regions of the mitral valve annulus in the apical four-chamber view [20]. The ratio of peak E-wave velocity and the average of peak e’-velocities (lateral and septal) was calculated as a non-invasive surrogate parameter of LV filling pressure (E/e’-ratio).

**Fig. 1:**
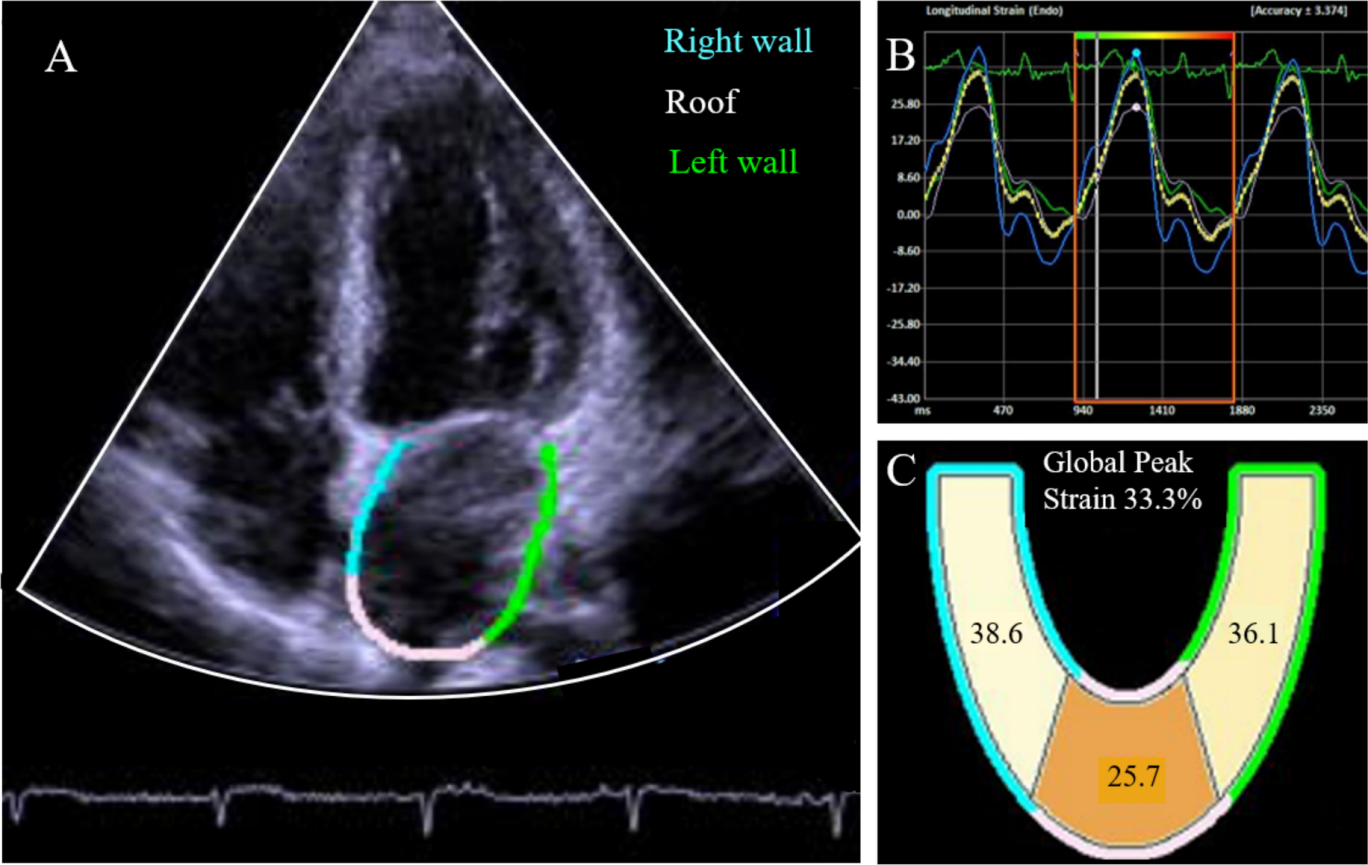
Exemplary measurement of left atrial global peak strain. Panel A: Apical 4-chamber view delineating the endocardial boundary of the left atrium in three sections (right wall, roof and left wall) as indicated by the respective colors. Panel B: Exemplary left atrial endocardial longitudinal strain curve derived from 2-D speckle-tracking. Panel C: Exemplary left atrial peak strain values for the three* different sections and derived left atrial global peak strain value*

The global peak strain of the left atrium was measured by three different investigators using 2-D speckle tracking. The quality of each echocardiographic image and analysis was evaluated on a scale from 1 to 4 (1: no breathing, well-defined, correct angle; 2: no breathing, angle not quite optimal and/or more difficult to define; 3: respiration and/or very poorly defined but plausible curve; 4: heavy breathing and/or no legitimate curve). We excluded all participants who were rated as 4 due to insufficient image quality to perform strain analysis. After optimal positioning of the myocardial search region in the apical four-chamber view, the automatic contour detection process of the myocardium was started in real time (Fig. 1). However, in some cases, the endo- and epicardial detected frames did not cover the entire endocardium and epicardium in real time so that they had to be corrected manually. The QRS complex was used as the initiation of the strain calculation (R-R gating). The software Siemens syngo SC2000 Version 4.0 (Siemens Healthineers, Erlangen, Germany) was used to evaluate and quantify echocardiography data from certified investigators in a single reading center. All examiners were blinded to the subjects' clinical information.

### Neuropsychological assessment

Cognitive function was assessed using the Animal Naming Test (ANT), the Trail Making Test and a 10-Word Learning Test. The ANT is a semantic verbal fluency test in which the individuals are asked to list as many names of animals as possible within one minute. All responses are counted excluding repetitions and non-animal words. The Trail Making Test consists of two parts, which reflect different areas of the human cognitive function. During part A (TMT-A), the individuals are asked to connect all numbers from 1 to 25 on a paper in sequential order, testing the individual’s information processing speed. A successful completion of Part B (TMT-B) requires the connection of all dots from 1 to 13 and letters from A to L while alternating letters and numbers (as in 1-A-2-B-3-C…), assessing attention and executive function. In both cases, TMT-A and TMT-B, the time to completion of the test is recorded as the test result, with time limits of 180 s and 360 s for TMT-A and TMT-B, respectively. For the 10-Word Learning Test, ten non-associated words were read to the participants in a different order in three rounds. Then participants were asked to freely recall these ten words after each round gaining one point for every correct response, and the sum of correct answers from all three rounds was calculated (immediate recall, range 0–30). In addition, participants were again asked to reproduce the ten words after 5–10 min (delayed recall, range 0–10). This test assesses the memory function.

### Cerebral magnetic resonance imaging

A subgroup of participants with present cardiovascular risk factors underwent standardized cMRI using a 3-T Siemens Skyra MRI scanner (Siemens, Erlangen, Germany; *N* = 2069). Further details of the cerebral magnetic resonance imaging protocol in the HCHS have been described in previous publications [21]. In addition to signs of infarct residuals, WMH were segmented on T1-weighted and FLAIR images with FSL’s Brain Intensity Abnormality Classification Algorithm (BIANCA) [22] and Locally Adaptive Threshold Estimation (LOCATE) [23] as described in detail previously. WMH were further divided into periventricular (pWMH) and deep (dWMH) by a 10 mm distance threshold to the ventricles [24–26]. WMH load was calculated as the proportion of WMH to brain tissue volume (intracranial volume—ventricle volume) and logarithmized for further statistical analysis based on a right-skewed distribution. Logarithmic pWMH and dWMH load were calculated analogously.

### Statistical analysis

Categorical variables are given as absolute and relative frequencies, continuous variables as median (25th/75th percentile). We used multiple imputation to handle missing information on clinical covariates (information on missing values and imputations are provided in Supplementary Table 1). We performed linear regression analyses with incremental adjustment to examine the association of echocardiographic parameters with the performance on each of the neuropsychological tests. In a first step, we calculated age- and sex-adjusted regression analyses (model 1). In multi-variable-adjusted analyses, arterial hypertension, body mass index, total serum cholesterol concentration, diabetes mellitus, current smoking, AF, prior/known myocardial infarction, heart failure and educational level were added as additional covariates (model 2). In a third and final step, analyses were additionally adjusted for further echocardiographic parameters of the left ventricle (LVEF and end-diastolic thickness of the interventricular septum; model 3). In sensitivity analyses, we excluded individuals with known AF, heart failure, and prior myocardial infarction. A two-sided *p*-value ≤ 0.05 was considered statistically significant for all analyses. Analyses were performed with R v.4.0.3 (www.R-project.org).

## Results

### Population characteristics

**Fig. 2:**
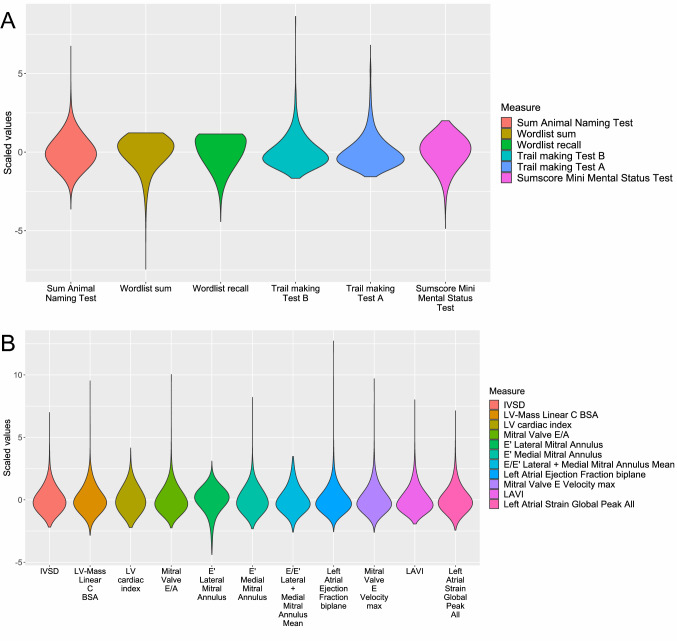
Violin plots showing the distribution of cognitive function test results (Panel A) and echocardiographic measures (Panel B) in the cohort. *BSA* body surface area; *IVSD* interventricular septal thickness; *LAVI* left atrial volume index; *LV* left ventricular

Overall, 7,852 individuals were analyzed (51.6% female, median age 62.0 [25th/75th percentile 55/69] years, Table 1). Diabetes was present in 596 individuals (7.6%), arterial hypertension in 5012 (63.8%), 1539 were current smokers (19.6%). AF was prevalent in 410 individuals (5.2%), HF was prevalent in 281 individuals (3.6%) and 218 individuals had a previous myocardial infarction (2.8%). The median left ventricular ejection fraction was 59% (25th/75th percentile 56%/62%), median left atrial volume index (LAVI) was 25 ml/m^2^ (25th/75th percentile 20 ml/m^2^ /30 ml/m^2^) and median LA global peak strain was 38% (25th/75th percentile 30%/48%). See Fig. 2 for the distribution of all cognitive function test results and echocardiographic measures in the cohort.

**Table 1:** Characteristics of the study population (*N* = 7852)

Clinical characteristics and cardiovascular risk factors/diseases	
Age (years)	62.0 (55.0; 69.0)
Female	4052 (51.6)
Education	
Low	398 (5.1)
Medium	3921 (50.0)
High	3533 (45.0)
Current smoker	1539 (19.6)
Body mass index (kg/m^2^)	25.8 (23.4; 28.9)
Arterial hypertension	5012 (63.8)
Diabetes mellitus	596 (7.6)
Total cholesterol (mg/dl)	209 (182; 237)
Atrial fibrillation	410 (5.2)
Prior myocardial infarction	218 (2.8)
Heart failure	281 (3.6)
Echocardiographic characteristics	
Left ventricular ejection fraction (%)	58.5 (55.5; 61.8)
Interventricular septal diameter (mm)	9.8 (8.8; 11.0)
E-wave-velocity (cm/s)	67.3 (57.1; 79.1)
E/A ratio	0.9 (0.8; 1.2)
e’-lateral velocity (cm/s)	10.2 (8.3; 12.3)
e’-septal velocity (cm/s)	8.4 (7.0; 10.1)
E/e’(mean) ratio	7.2 (6.1; 8.5)
Left atrial volume index (ml/m^2^)	25.2 (20.4; 30.8)
Left atrial ejection fraction (%)	49.2 (42.9; 54.8)
Left atrial global peak strain (%)	38.0 (29.9; 48.3)
Cognitive tests	
Animal naming test	25 (20, 30)
Trail making test part A (s)	37 (29, 48)
Trail making test part B (s)	79.7 (61.0; 106.5)
Word list immediate recall	23.0 (20.0, 25.0)
Word list delayed recall	8.0 (7.0, 9.0)
Cranial magnetic resonance imaging	
White matter hyperintensity volume (ml)	1.47 (0.76, 2.95)
White matter hyperintensity load (%)	0.10 (0.05, 0.20)

Categorical variables are presented as absolute and relative frequencies, continuous variables as median (25th and 75th quartile)

### Association between echocardiographic characteristics and cognitive function

**Fig. 3:**
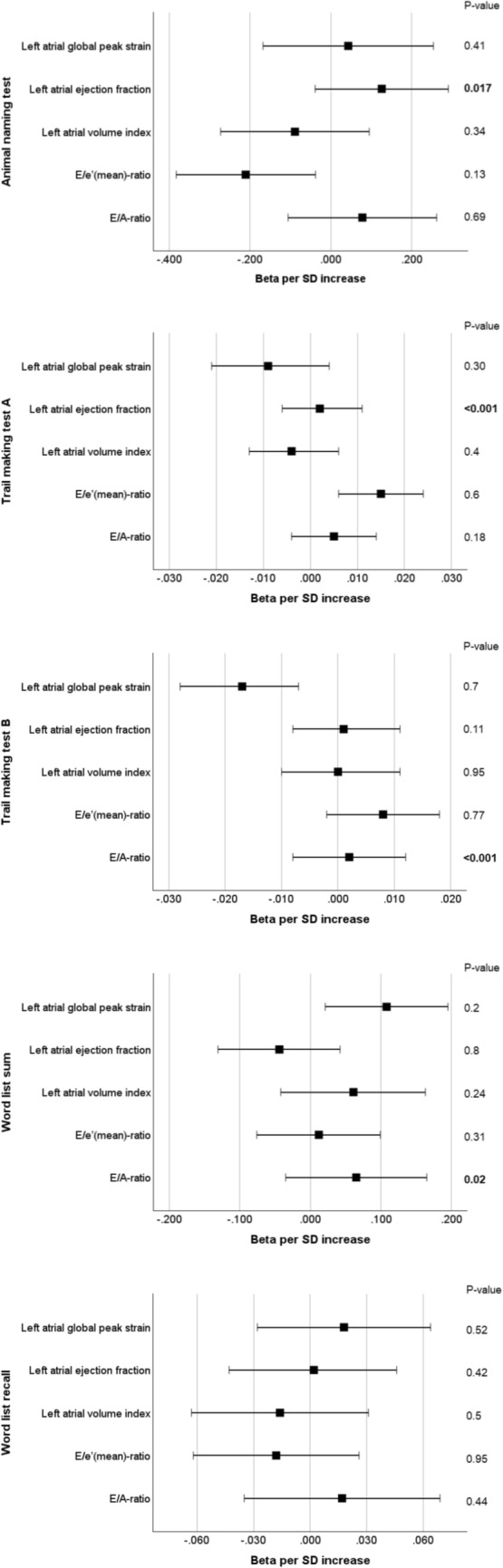
Association of echocardiographic parameters with the performance on the neuropsychological tests using multi-variable adjusted linear regression analysis (model 3, adjusted for age, sex, arterial hypertension, body mass index, total serum cholesterol concentration, diabetes mellitus, current smoking, AF, prior/known myocardial infarction, heart failure and educational level, left ventricular ejection fraction and end-diastolic thickness of the interventricular septum). The beta per standard deviation (SD) increase is given as a box with whiskers indicating the 95% confidence interval. The *p* values are given on the right column, significant results (two-sided *p*-value ≤ 0.05) are printed in bold

Age- and sex-adjusted regression models (model 1) revealed differential associations between echocardiographic parameters and distinct domains of cognitive function (Table 2). After adjustment for clinical covariates (model 2), the E/e’-ratio remained significantly associated with the level of impairment in the Animal Naming Test (beta − 0.185 per one standard deviation [SD] increase, 95% confidence interval [CI] − 0.36-[− 0.01]) and the Trail Making Test part A (0.01, 95% CI 0.003–0.02). Left atrial peak global strain was significantly associated with a positive performance on the Trail Making Test part A (− 0.01, 95% CI − 0.02-[− 0.002]), the Trail Making Test part B (− 0.02, 95% CI − 0.03-[− 0.01]) and the immediate recall part of the 10-Word Learning Test (0.11 per one SD increase, 95% CI 0.02–0.20). The E/A-ratio, LAVI and the LA ejection fraction were not significantly associated with cognitive test results in multi-variable-adjusted regression analyses (Fig. 3). None of the echocardiographic parameters were associated with the performance on the delayed recall part of the 10-Word Learning Test. Further adjustment for LVEF and septal wall thickness (model 3) did not alter the significance of these results. These findings were robust in sensitivity analyses excluding individuals with AF and individuals with known heart failure (Supplementary Table 2) or prior myocardial infarction (Supplementary Table 3).

**Table 2:** Associations of echocardiographic characteristics with performance on cognitive tests

	Model 1	Model 2	Model 3			
	Beta per SD increase (95% CI)	*p*-value	Beta per SD increase (95% CI)	*p*-value	Beta per SD increase (95% CI)	*p*-value
Animal naming test						
E/A ratio	0.22 (0.051, 0.389)	**0.01**	0.076 (– 0.106, 0.259)	0.41	0.078 (– 0.106, 0.262)	0.41
E/e’(mean) ratio	– 0.362 (– 0.532, – 0.193)	** < 0.001**	– 0.185 (– 0.355, – 0.014)	**0.03**	– 0.211 (– 0.383, – 0.038)	**0.017**
Left atrial volume index	– 0.027 (– 0.206, 0.152)	0.77	– 0.093 (– 0.277, 0.091)	0.32	– 0.089 (– 0.273, 0.095)	0.34
Left atrial ejection fraction	0.118 (– 0.04, 0.275)	0.14	0.151 (– 0.013, 0.315)	0.07	0.126 (– 0.039, 0.291)	0.13
Left atrial global peak strain	0.157 (– 0.052, 0.365)	0.14	0.055 (– 0.152, 0.263)	0.60	0.043 (– 0.168, 0.254)	0.69
Trail making test A						
E/A ratio	– 0.003 (– 0.011, 0.006)	0.53	0.006 (– 0.004, 0.015)	0.23	0.005 (– 0.004, 0.014)	0. 30
E/e’(mean) ratio	0.019 (0.011, 0.028)	** < 0.001**	0.014 (0.005, 0.023)	**0.002**	0.015 (0.006, 0.024)	**0.001**
Left atrial volume index	– 0.003 (– 0.012, 0.006)	0.54	– 0.003 (– 0.012, 0.007)	0.55	– 0.004 (– 0.013, 0.006)	0.4
Left atrial ejection fraction	– 0.002 (– 0.01, 0.006)	0.65	0.001 (– 0.008, 0.009)	0.87	0.002 (– 0.006, 0.011)	0.60
Left atrial global peak strain	– 0.011 (– 0.023, 0.001)	0.08	– 0.008 (– 0.021, 0.004)	0.18	– 0.009 (– 0.021, 0.004)	0.18
Trail making test B						
E/A ratio	– 0.012 (– 0.021, – 0.002)	**0.02**	0 (– 0.01, 0.01)	0.94	0.002 (– 0.008, 0.012)	0.70
E/e’(mean) ratio	0.019 (0.009, 0.029)	** < 0.001**	0.008 (– 0.002, 0.018)	0.13	0.008 (– 0.002, 0.018)	0.11
Left atrial volume index	– 0.002 (– 0.012, 0.007)	0.63	– 0.001 (– 0.012, 0.01)	0.84	0 (– 0.01, 0.011)	0.95
Left atrial ejection fraction	0 (– 0.009, 0.009)	0.95	0.002 (– 0.008, 0.011)	0.70	0.001 (– 0.008, 0.011)	0.77
Left atrial global peak strain	– 0.024 (– 0.034, – 0.013)	** < 0.001**	– 0.019 (– 0.029, – 0.008)	**0.001**	– 0.017 (– 0.028, – 0.007)	**0.001**
Word list sum						
E/A ratio	0.101 (0.01, 0.191)	**0.03**	0.064 (– 0.036, 0.163)	0.21	0.065 (– 0.035, 0.165)	0.20
E/e’(mean) ratio	– 0.071 (– 0.157, 0.015)	0.1	0.008 (– 0.078, 0.095)	0.85	0.012 (– 0.076, 0.099)	0.8
Left atrial volume index	0.088 (– 0.006, 0.182)	0.07	0.06 (– 0.041, 0.16)	0.24	0.061 (– 0.042, 0.163)	0.24
Left atrial ejection fraction	– 0.054 (– 0.135, 0.028)	0.2	– 0.047 (– 0.132, 0.039)	0.28	– 0.044 (– 0.131, 0.042)	0.31
Left atrial global peak strain	0.146 (0.06, 0.231)	**0.001**	0.105 (0.017, 0.192)	**0.02**	0.108 (0.021, 0.195)	**0.02**

	Model 1	Model 2	Model 3			
	Beta per SD increase (95% CI)	*p*-value	Beta per SD increase (95% CI)	*p*-value	Beta per SD increase (95% CI)	*p*-value
Word list recall						
E/A ratio	0.03 (– 0.016, 0.077)	0.20	0.022 (– 0.03, 0.074)	0.41	0.017 (– 0.035, 0.069)	0.52
E/e’(mean) ratio	– 0.042 (– 0.085, 0)	0.05	– 0.019 (– 0.063, 0.024)	0.38	– 0.018 (– 0.062, 0.026)	0.42
Left atrial volume index	0.008 (– 0.035, 0.052)	0.71	– 0.011 (– 0.057, 0.036)	0.65	– 0.016 (– 0.063, 0.031)	0.50
Left atrial ejection fraction	– 0.004 (– 0.046, 0.038)	0.85	– 0.003 (– 0.047, 0.042)	0.90	0.002 (– 0.043, 0.046)	0.95
Left atrial global peak strain	0.039 (– 0.006, 0.083)	0.09	0.021 (– 0.024, 0.066)	0.36	0.018 (– 0.028, 0.064)	0.44

Model 1: Linear regression analyses adjusted for age and sex; Model 2: Model 1 with additional adjustment for education level and cardiovascular risk factors/diseases (arterial hypertension, diabetes mellitus, current smoking, body mass index, total cholesterol, heart rate, atrial fibrillation, prior myocardial infarction, and heart failure); Model 3: Model 2 with additional adjustment for left ventricular ejection fraction and interventricular septum thickness

P-values reaching the statistical significance threshold of <0.05 are marked in bold

### Association between echocardiographic characteristics and white matter hyperintensities

The E/e’-ratio was positively associated with the total and periventricular WMH load in age- and sex-adjusted regression analyses (Table 3). These associations were no longer evident after adjustment for clinical covariates. All other echocardiographic parameters showed no significant associations with WMH load. Deep WMH load did not show significant associations with any echocardiographic parameter regardless of adjustments.

**Table 3:** Associations of echocardiographic characteristic with white matter hyperintensities von cranial magnetic resonance imaging (*N* = 1918)

	Model 1	Model 2	Model 3			
	Beta per SD increase (95% CI)	*p*-value	Beta per SD increase (95% CI)	*p*-value	Beta per SD increase (95% CI)	*p*-value
White matter hyperintensities load						
E/A ratio	– 0.029 (– 0.075, 0.018)	0.23	– 0.016 (– 0.067, 0.035)	0.54	– 0.015 (– 0.066, 0.037)	0.57
E/e’(mean) ratio	0.063 (0.016, 0.11)	**0.009**	0.038 (– 0.01, 0.086)	0.12	0.039 (– 0.009, 0.088)	0.11
Left atrial volume index	– 0.027 (– 0.073, 0.02)	0.26	– 0.01 (– 0.058, 0.038)	0.68	– 0.009 (– 0.057, 0.039)	0.72
Left atrial ejection fraction	0.008 (– 0.036, 0.052)	0.72	0.009 (– 0.037, 0.054)	0.71	0.009 (– 0.036, 0.055)	0.69
Left atrial global peak strain	– 0.03 (– 0.09, 0.029)	0.31	– 0.012 (– 0.075, 0.051)	0.70	– 0.01 (– 0.074, 0.053)	0.75
Deep white matter hyperintensities load						
E/A ratio	– 0.025 (– 0.12, 0.069)	0.60	– 0.032 (– 0.136, 0.072)	0.55	– 0.029 (– 0.133, 0.075)	0.59
E/e’(mean) ratio	0.033 (– 0.067, 0.132)	0.52	0.02 (– 0.083, 0.122)	0.70	0.029 (– 0.073, 0.132)	0.58
Left atrial volume index	0.004 (– 0.092, 0.101)	0.93	– 0.003 (– 0.104, 0.099)	0.96	0.001 (– 0.101, 0.103)	0.98
Left atrial ejection fraction	– 0.026 (– 0.115, 0.064)	0.57	– 0.015 (– 0.108, 0.079)	0.76	– 0.007 (– 0.101, 0.087)	0.88
Left atrial global peak strain	– 0.035 (– 0.16, 0.09)	0.57	– 0.042 (– 0.172, 0.088)	0.52	– 0.033 (– 0.164, 0.097)	0.61
Periventricular white matter hyperintensities load						
E/A– ratio	– 0.037 (– 0.084, 0.011)	0.13	– 0.02 (– 0.072, 0.032)	0.46	– 0.019 (– 0.072, 0.034)	0.48
E/e’(mean) ratio	0.071 (0.023, 0.119)	**0.003**	0.043 (– 0.006, 0.091)	0.09	0.043 (– 0.005, 0.092)	0.08
Left atrial volume index	– 0.033 (– 0.08, 0.014)	0.17	– 0.015 (– 0.064, 0.033)	0.54	– 0.014 (– 0.063, 0.035)	0.57
Left atrial ejection fraction	0.008 (– 0.037, 0.053)	0.73	0.008 (– 0.038, 0.054)	0.74	0.008 (– 0.039, 0.054)	0.74
Left atrial global peak strain	– 0.029 (– 0.086, 0.028)	0.31	– 0.008 (– 0.068, 0.052)	0.80	– 0.007 (– 0.067, 0.054)	0.83

Model 1: Linear regression analyses adjusted for age and sex; Model 2: Model 1 with additional adjustment for education level and cardiovascular risk factors/diseases (arterial hypertension, diabetes mellitus, current smoking, body mass index, total cholesterol, heart rate, atrial fibrillation, prior myocardial infarction, and heart failure); Model 3: Model 2 with additional adjustment for left ventricular ejection fraction and interventricular septum thickness

P-values reaching the statistical significance threshold of <0.05 are marked in bold

## Discussion

In this cross-sectional all-comers cohort, we have identified relations of echocardiographic parameters of atrial cardiomyopathy with cognitive performance. We describe an association of lower left atrial strain, an early echocardiographic sign of atrial dysfunction related to atrial cardiomyopathy, with reduced performance on neurocognitive tests. Similarly, diastolic ventricular impairment was associated with cognitive function. We observed a significant association between the E/e’-ratio, an established parameter in the diagnosis of LV diastolic dysfunction, and verbal fluency as well as information processing speed. LA global peak strain was associated with processing speed, executive function, and immediate memory function. These associations remained robust after adjustment for parameters of LV systolic function and cardiac structure and appeared to be slightly stronger in individuals without AF.

Whereas the E/e’-ratio was positively associated with the total and periventricular WMH load in age- and sex-adjusted regression analyses, echocardiographic characteristics were not associated with the extent of WMH load on cMRI after comprehensive adjustment for clinical covariates.

Prior studies on the association of echocardiographic parameters of LV diastolic and LA function with cognitive function have yielded inconclusive results. In the population-based Hoorn Study, the authors found that the E/e’-ratio was not associated with any domain of cognitive function, whereas LAVI was associated with information processing speed, attention and executive functioning [27]. In a more recent population-based study, LA diameter indexed to the body surface area was significantly associated with the working memory only, but not with the ANT [28]. The E/e’-ratio, but not LAVI, was found to be predictive of executive functioning and visuo-constructional abilities in 373 individuals with chronic heart disease other than heart failure [29]. Diastolic dysfunction was associated with incident dementia in the population-based Rotterdam study [4]. In individuals with chronic heart disease, diastolic dysfunction was a predictor of executive functioning and visuo-constructional abilities [29]. Furthermore, parameters of LV hypertrophy, which is closely linked to diastolic dysfunction, have been shown to be associated with an increased risk of cognitive impairment [30].

The diverging results between studies regarding the association of echocardiographic parameters with cognitive function might be partly explained by differences in study populations and methods, particularly in the assessment of cognitive function, but also of echocardiographic parameters. In addition, the absence of a significant association of the E/e’-ratio with cognitive function in some prior studies might also be explained by a lack of statistical power considering that the number of included individuals was comparatively low and the associations at the population level are rather weak.

Left atrial function and morphology are closely related to LV (diastolic) function, e.g., LAVI is considered a surrogate parameter for the chronicity and severity of LV diastolic dysfunction and also considered a key marker for atrial cardiomyopathy [8, 20, 31, 32]. The assessment of LAVI has been shown to be superior to LA diameter or area regarding the prediction of future cardiovascular events [33]. However, LA dilatation occurs in a more advanced stage of diastolic dysfunction compared to alterations in LA strain [31]. Furthermore, LA strain seems to correspond to the degree of atrial fibrosis on cardiac magnetic resonance imaging and might therefore indicate LA remodeling [34–36]. Therefore, the observed association of LA strain with the performance on the cognitive tests might be explained by its higher sensitivity for subtle changes in LV diastolic and atrial function in this comparatively healthy study population.

Impairment in executive function, processing speed and verbal fluency are commonly found in individuals with vascular brain lesions [1]. Common vascular causes for cognitive impairment and dementia are ischemic brain lesions and cerebral small vessel disease [1]. As AF is known to be associated with incident dementia, a possible pathophysiologic explanation for our findings might be a mediation through paroxysmal, asymptomatic AF with possible subsequent clinically silent brain infarctions through cardiac embolism [3, 35]. It has been proposed that a thrombogenic atrial substrate due to various alterations in (left) atrial morphology and function could contribute to atrial thromboembolism even in the absence of (clinically overt) AF [1, 6, 9, 10, 20]. However, we did not observe a significant association between E/e’-ratio, LAVI and LA strain with the presence of WMH in the subgroup with available cMRI after adjustment for clinical covariates (Table 3). The link between left ventricular diastolic function and left atrial cardiomyopathy with cognitive dysfunction might also be explained as a consequence of chronic cerebral hypoperfusion due to a decrease in cardiac output, particularly during periods of higher circulatory demand resulting in acute dysfunction and chronic metabolic dysregulation of neuronal tissue [1, 9, 10]. Other mechanisms have been proposed as links between these conditions, such as impaired cerebral autoregulation as well as capillary dysfunction and disruption of the blood–brain barrier [37, 38]. Furthermore, cerebral small vessel disease, LV diastolic dysfunction and atrial cardiomyopathy share underlying risk factors such as longstanding hypertension or diabetes mellitus [8, 13]. Both cerebral hypoperfusion and small vessel disease possibly lead to ischemia of the brain tissue. WMH are an imaging marker of cerebral small vessel disease and assumably result from (chronic) ischemia [39]. Their incidence and progression have been linked to a decline in cognitive function [13, 40]. Left atrial diameter was associated with prevalent, but not with incident brain infarcts or WMH detected by cMRI in the Cardiovascular Health Study [41]. In an earlier community-based study, an increasing minimum LA volume index and a decrease in LA ejection fraction, but not (maximum) LAVI were significantly associated with silent brain infarction and WMH [42]. In contrast, two other studies could not find associations between AF and WMH [43, 44]. We did not detect any significant associations between the echocardiographic parameters studied and WMH, possibly indicating that the association between these parameters and cognitive function is not primarily explained by structural changes resulting from progression of cerebral small vessel disease. Alternatively, these structural brain changes might be more subtle than WMH, and more sensitive measures than WMH might be able to detect such minute changes in future.

### Limitations and strengths

Some limitations merit consideration. This study should be interpreted as exploratory, given that multiple predictors were assessed and adjustment for multiple testing was not performed. We examined a relatively cognitive healthy cohort, which could possibly weaken the observed associations. Given the often paroxysmal and asymptomatic nature of AF, some cases of AF might have been missed and therefore could have influenced the observed associations. However, AF diagnosis was based on history, study ECG and 7-day Holter monitoring which was available in a subset of participants. The presence of AF during the examinations could potentially have influenced the results of the neuropsychological assessment and limits the informative value of the echocardiography, and the examinations were not repeated to exclude this effect. However, we performed a sensitivity analysis to account for this limitation, and the results remained robust. cMRI was only available in a subgroup and our study might have been underpowered to detect subtle association between cardiac function and WMH. However, in our large community-based cohort with standardized data acquisition, in-depth phenotyping and careful adjustment of analyses, we can present robust evidence on the relationship between left ventricular and atrial function with cognitive performance.

## Conclusion

In our large population-based cohort, including more than 7000 individuals, we report significant associations of subtle changes in echocardiographic characteristics of left ventricular diastolic and LA function with measurable differences in the performance across a broad spectrum of cognitive tests and cerebral imaging. In particular, LA strain seems to be a promising parameter for detection of early changes in cardiac function associated with changes in different domains of cognition. Our study relates easily quantifiable cardiac parameters with cognitive impairment. Validation of these results in external studies could yield additional insights into the underlying pathophysiological mechanisms of atrial impairment with cerebral pathologies. Our better understanding of the two increasingly common conditions may have significant impact on clinical medicine and public health, possibly enabling early identification of patients at risk for prevention in future.

## Supplementary Information

Below is the link to the electronic supplementary material.

Supplementary file1 (DOCX 33 kb)

## Data Availability

The datasets used and/or analysed during the current study available from the corresponding author on reasonable request.

## Abbreviations

AF: Atrial fibrillation
ANT: Animal naming test
CI: Confidence interval
cMRI: Cerebral magnetic resonance imaging
HCHS: Hamburg City Health Study
ISCED: International Standard Classification of Education
LA: Left atrial
LAVI: Maximum left atrial volume index
LV: Left ventricular
LVEF: Left ventricular ejection fraction
SD: Standard deviation
TMT-A: Trail Making Test part A
TMT-B: Trail Making Test part B
(d/p) WMH: (Deep/periventricular) white matter hyperintensities
