# Supplementary material for: Subtle signs of atrial cardiomyopathy and left ventricular diastolic dysfunction are associated with reduced cognitive function: results from the Hamburg City Health Study
Source: Clin Res Cardiol. 2024 Nov 27;114(12):1658–70. doi: 10.1007/s00392-024-02581-5 (PMC12708828; doi:10.1007/s00392-024-02581-5)
Supplement: Supplementary file 1 — Supplementary file1 (DOCX 33 kb) [file 392_2024_2581_MOESM1_ESM.docx]

**Reduced cognitive function is associated with subtle signs of atrial cardiomyopathy and left ventricular diastolic dysfunction – results from the Hamburg City Health Study**

Ohlrogge et al.

*Supplementary Material*

**Supplementary Table 1.** Comparisons of complete case and imputed data of the study population

**Supplementary Table 2.** Associations of echocardiographic characteristics with cognitive function in individuals without atrial fibrillation

**Supplementary Table 3.** Associations of echocardiographic characteristics with cognitive function in individuals without prior myocardial infarction or heart failure

| **Supplementary Table 1.** **Comparisons of complete case and imputed data of the study population** | | | |  |
| --- | --- | --- | --- | --- |
|  | **Available cases** | **Imputed data** | **Missings** | |
| **Clinical characteristics** | | | | |
| Age (Years) | 63 (55, 69) | 63 (55, 69) | 0 (0) | |
| Education |  |  | 449 (5.7) | |
| Short | 353 (4.8) | 398 (5.1) |  | |
| Medium | 3687 (49.8) | 3921 (50.0) |  | |
| High | 3363 (45.4) | 3533 (45.0) |  | |
| Atrial fibrillation No. (%) | 410 (5.2) | 410 (5.2) | 790 (10.1) | |
| Prior myocardial infarction No. (%) | 218 (2.8) | 218 (2.8) | 55 (0.7) | |
| Heart failure No. (%) | 281 (3.6) | 281 (3.6) | 891 (11.3) | |
| Dementia No. (%) | 15 (0.2) | 15 (0.2) | 29 (0.4) | |
| Current smoker No. (%) | 1530 (19.6) | 1539 (19.6) | 43 (0.5) | |
| Body mass index (kg/m²) | 26.0 (23.4, 29.1) | 25.8 (23.4, 28.9) | 445 (5.7) | |
| Arterial hypertension No. (%) | 4888 (65.5) | 5012 (63.8) | 389 (5) | |
| Antihypertensive drugs No. (%) | 2437 (32.6) | 2524 (32.2) | 375 (4.8) | |
| Diabetes mellitus No. (%) | 588 (8.1) | 596 (7.6) | 621 (7.9) | |
| Total cholesterol (mg/dl) | 209 (182, 237) | 209 (182, 237) | 227 (2.9) | |
| **Echocardiographic parameters** | | | | |
| Left ventricular ejection fraction (%) | 58.5 (55.5, 61.8) | 58.5 (55.5, 61.8) | 623 (7.9) | |
| Interventricular septal diameter (mm) | 9.8 (8.8, 11.0) | 9.8 (8.8, 11.0) | 673 (8.6) | |
| E-wave-velocity (cm/s) | 67.3 (57.1, 79.0) | 67.3 (57.1, 79.1) | 514 (6.5) | |
| E/A-ratio | 0.9 (0.8, 1.2) | 0.9 (0.8, 1.2) | 611 (7.8) | |
| e’-lateral velocity (cm/s) | 10.2 (8.3, 12.3) | 10.2 (8.3, 12.3) | 560 (7.1) | |
| e’-septal velocity (cm/s) | 8.4 (7.0, 10.1) | 8.4 (7.0, 10.1) | 649 (8.3) | |
| E/e’(mean)-ratio | 7.2 (6.0, 8.5) | 7.2 (6.1, 8.5) | 886 (11.3) | |
| Left atrial volume index (ml/m²) | 25.3 (20.3, 30.9) | 25.2 (20.4, 30.8) | 1137 (14.5) | |
| Left atrial ejection fraction (%) | 49.0 (42.9, 54.6) | 49.2 (42.9, 54.8) | 2089 (26.6) | |
| Left atrial global peak strain (%) | 37.7 (29.8, 47.7) | 38.0 (29.9, 48.3) | 2778 (35.4) | |
| **Cognitive tests *** | | | | |
| Animal Naming Test | 25 (20, 30) | 25 (20, 30) | 531 (6.8) | |
| Trail Making Test A | 37 (29, 47) | 37 (29, 48) | 835 (10.6) | |
| Trail Making Test B | 79 (61, 103) | 79.7 (61.0, 106.5) | 954 (12.1) | |
| Word List Immediate Recall | 23.0 (20.0, 25.0) | 23.0 (20.0, 25.0) | 708 (9) | |
| Word List Delayed Recall | 8.0 (7.0, 9.0) | 8.0 (7.0, 9.0) | 932 (11.9) | |
| **Cranial magnetic resonance imaging *** | | | | |
| White Matter Hyperintensity Volume (ml) | 1.47 (0.76, 2.95) | 1.47 (0.76, 2.95) | 5932 (75.5) | |
| White Matter Hyperintensity Load (%) | 0.10 (0.05, 0.20) | 0.10 (0.05, 0.20) | 5934 (75.6) | |
| * Outcome variables (cognitive test results and white matter hyperintensities on cerebral magnetic resonance imaging) were not imputed; categorical variables are presented as absolute and relative frequencies, continuous variables as median (25th/75th percentile). | | | | |

| **Supplementary Table 2. Associations of echocardiographic characteristics with cognitive function in individuals without atrial fibrillation** | | | | | | | | | | | | |  |
| --- | --- | --- | --- | --- | --- | --- | --- | --- | --- | --- | --- | --- | --- |
|  | **Model 1** | | | | **Model 2** | | | | **Model 3** | | | |  |
|  | **Beta per SD increase (95% CI)** | | **p-value** | | **Beta per SD increase (95% CI)** | | **p-value** | | **Beta per SD increase (95% CI)** | | **p-value** | |  |
| **Animal naming test** | | | | | | | | | | | | |  |
| E/A-ratio | 0.297 (0.118, 0.476) | | 0.001 | | 0.084 (-0.107, 0.275) | | 0.39 | | 0.087 (-0.105, 0.28) | | 0.37 | |  |
| E/e’(mean)-ratio | -0.382 (-0.557, -0.207) | | <0.001 | | -0.209 (-0.385, -0.033) | | **0.02** | | -0.232 (-0.409, -0.054) | | **0.011** | |  |
| Left atrial volume index | -0.094 (-0.278, 0.09) | | 0.32 | | -0.117 (-0.297, 0.062) | | 0.20 | | -0.117 (-0.297, 0.062) | | 0.2 | |  |
| Left atrial ejection fraction | 0.148 (-0.016, 0.311) | | 0.08 | | 0.154 (-0.014, 0.322) | | 0.07 | | 0.135 (-0.034, 0.304) | | 0.12 | |  |
| Left atrial global peak strain | 0.118 (-0.091, 0.327) | | 0.27 | | 0.036 (-0.17, 0.241) | | 0.73 | | 0.023 (-0.185, 0.231) | | 0.83 | |  |
| **Trail making test A** | | | | | | | | | | | | |  |
| E/A-ratio | -0.002 (-0.01, 0.007) | | 0.71 | | 0.008 (-0.002, 0.017) | | 0.11 | | 0.007 (-0.003, 0.016) | | 0.16 | |  |
| E/e’(mean)-ratio | 0.019 (0.01, 0.027) | | <0.001 | | 0.012 (0.003, 0.021) | | **0.007** | | 0.013 (0.004, 0.022) | | **0.003** | |  |
| Left atrial volume index | -0.004 (-0.013, 0.005) | | 0.32 | | -0.004 (-0.013, 0.005) | | 0.37 | | -0.005 (-0.014, 0.004) | | 0.27 | |  |
| Left atrial ejection fraction | -0.001 (-0.01, 0.007) | | 0.72 | | 0.001 (-0.007, 0.009) | | 0.81 | | 0.003 (-0.006, 0.011) | | 0.53 | |  |
| Left atrial global peak strain | -0.013 (-0.022, -0.004) | | 0.003 | | -0.01 (-0.019, -0.002) | | **0.02** | | -0.01 (-0.019, -0.002) | | **0.018** | |  |
| **Trail making test B** | | | | | | | | | | | | |  |
| E/A-ratio | -0.015 (-0.025, -0.005) | | 0.003 | | 0 (-0.01, 0.011) | | 0.93 | | 0.002 (-0.009, 0.012) | | 0.73 | |  |
| E/e’(mean)-ratio | 0.02 (0.01, 0.031) | | <0.001 | | 0.009 (-0.001, 0.02) | | 0.08 | | 0.01 (-0.001, 0.02) | | 0.03 | |  |
| Left atrial volume index | 0.001 (-0.009, 0.011) | | 0.89 | | 0 (-0.01, 0.011) | | 0.97 | | 0.001 (-0.009, 0.012) | | 0.81 | |  |
| Left atrial ejection fraction | -0.003 (-0.012, 0.006) | | 0.52 | | 0.001 (-0.008, 0.01) | | 0.85 | | 0.001 (-0.009, 0.01) | | 0.86 | |  |
| Left atrial global peak strain | -0.022 (-0.032, -0.012) | | <0.001 | | -0.018 (-0.028, -0.008) | | **0.001** | | -0.017 (-0.027, -0.007) | | **0.001** | |  |
| **Word list sum** | | | | | | | | | | | | |  |
| E/A-ratio | 0.122 (0.034, 0.21) | | 0.007 | | 0.044 (-0.05, 0.138) | | 0.36 | | 0.047 (-0.048, 0.141) | | 0.33 | |  |
| E/e’(mean)-ratio | -0.071 (-0.158, 0.016) | | 0.11 | | 0.003 (-0.084, 0.091) | | 0.94 | | 0.005 (-0.083, 0.094) | | 0.91 | |  |
| Left atrial volume index | 0.073 (-0.017, 0.163) | | 0.11 | | 0.072 (-0.019, 0.163) | | 0.12 | | 0.075 (-0.017, 0.168) | | 0.11 | |  |
| Left atrial ejection fraction | -0.046 (-0.13, 0.038) | | 0.29 | | -0.057 (-0.144, 0.029) | | 0.19 | | -0.058 (-0.145, 0.029) | | 0.20 | |  |
| Left atrial global peak strain | 0.131 (0.043, 0.219) | | 0.004 | | 0.107 (0.019, 0.195) | | **0.018** | | 0.111 (0.023, 0.199) | | **0.013** | |  |
| **(Continued)** | | | | | | | | | | | | | |
|  | | **Model 1** | | | | **Model 2** | | | | **Model 3** | | | |
|  | | **Beta per SD increase (95% CI)** | | **p-value** | | **Beta per SD increase (95% CI)** | | **p-value** | | **Beta per SD increase (95% CI)** | | **p-value** | |
| **Word list recall** | | | | | | | | | | | | | |
| E/A-ratio | | 0.037 (-0.008, 0.082) | | 0.11 | | 0.01 (-0.039, 0.059) | | 0.69 | | 0.006 (-0.043, 0.054) | | 0.82 | |
| E/e’(mean)-ratio | | -0.044 (-0.088, 0) | | 0.05 | | -0.024 (-0.068, 0.02) | | 0.29 | | -0.024 (-0.068, 0.021) | | 0.30 | |
| Left atrial volume index | | -0.008 (-0.052, 0.036) | | 0.72 | | -0.011 (-0.054, 0.033) | | 0.63 | | -0.015 (-0.059, 0.029) | | 0.5 | |
| Left atrial ejection fraction | | -0.007 (-0.05, 0.035) | | 0.74 | | -0.018 (-0.061, 0.026) | | 0.43 | | -0.015 (-0.059, 0.029) | | 0.50 | |
| Left atrial global peak strain | | 0.034 (-0.011, 0.08) | | 0.14 | | 0.026 (-0.02, 0.071) | | 0.27 | | 0.023 (-0.023, 0.069) | | 0.33 | |
| Model 1: Cox regressions analyses adjusted for age and sex; Model 2: Model 1+ additional adjustment for education level and cardiovascular risk factors/diseases (arterial hypertension, diabetes mellitus, current smoking, body mass index, total cholesterol, heart rate, prior myocardial infarction, and heart failure); Model 3: Model 2 + additional adjustment for left ventricular ejection fraction and interventricular septum thickness | | | | | | | | | | | | | |

| **Supplementary Table 3. Associations of echocardiographic characteristics with cognitive function in individuals without prior myocardial infarction or heart failure** | | | | | | | | | | | | |  |
| --- | --- | --- | --- | --- | --- | --- | --- | --- | --- | --- | --- | --- | --- |
|  | **Model 1** | | | | **Model 2** | | | | **Model 3** | | | |  |
|  | **Beta per SD increase (95% CI)** | | **p-value** | | **Beta per SD increase (95% CI)** | | **p-value** | | **Beta per SD increase (95% CI)** | | **p-value** | |  |
| **Animal naming test** | | | | | | | | | | | | |  |
| E/A-ratio | 0.251 (0.074, 0.427) | | 0.006 | | 0.084 (-0.106, 0.273) | | 0.39 | | 0.085 (-0.107, 0.277) | | 0.39 | |  |
| E/e’(mean)-ratio | -0.348 (-0.521, -0.176) | | <0.001 | | -0.204 (-0.375, -0.032) | | **0.02** | | -0.225 (-0.397, -0.053) | | **0.01** | |  |
| Left atrial volume index | -0.074 (-0.255, 0.107) | | 0.42 | | -0.098 (-0.276, 0.081) | | 0.28 | | -0.098 (-0.279, 0.083) | | 0.29 | |  |
| Left atrial ejection fraction | 0.116 (-0.049, 0.28) | | 0.17 | | 0.111 (-0.059, 0.282) | | 0.2 | | 0.093 (-0.079, 0.265) | | 0.29 | |  |
| Left atrial global peak strain | 0.152 (-0.046, 0.35) | | 0.13 | | 0.083 (-0.114, 0.279) | | 0.41 | | 0.068 (-0.132, 0.268) | | 0.50 | |  |
| **Trail making test A** | | | | | | | | | | | | |  |
| E/A-ratio | -0.002 (-0.01, 0.007) | | 0.71 | | 0.008 (-0.002, 0.017) | | 0.11 | | 0.007 (-0.003, 0.016) | | 0.16 | |  |
| E/e’(mean)-ratio | 0.019 (0.01, 0.027) | | <0.001 | | 0.012 (0.003, 0.021) | | **0.007** | | 0.013 (0.004, 0.022) | | **0.003** | |  |
| Left atrial volume index | -0.004 (-0.013, 0.005) | | 0.35 | | -0.004 (-0.013, 0.005) | | 0.37 | | -0.005 (-0.014, 0.004) | | 0.27 | |  |
| Left atrial ejection fraction | -0.001 (-0.01, 0.007) | | 0.72 | | 0.001 (-0.007, 0.009) | | 0.81 | | 0.003 (-0.006, 0.011) | | 0.53 | |  |
| Left atrial global peak strain | -0.013 (-0.022, -0.004) | | 0.003 | | -0.01 (-0.019, -0.002) | | **0.02** | | -0.01 (-0.019, -0.002) | | **0.018** | |  |
| **Trail making test B** | | | | | | | | | | | | |  |
| E/A-ratio | -0.013 (-0.023, -0.003) | | 0.01 | | 0.001 (-0.009, 0.012) | | 0.78 | | 0.003 (-0.007, 0.014) | | 0.54 | |  |
| E/e’(mean)-ratio | 0.02 (0.01, 0.029) | | <0.001 | | 0.01 (0, 0.02) | | **0.045** | | 0.01 (0.001, 0.02) | | **0.039** | |  |
| Left atrial volume index | 0 (-0.011, 0.011) | | 0.96 | | 0 (-0.011, 0.011) | | 0.98 | | 0.002 (-0.01, 0.013) | | 0.77 | |  |
| Left atrial ejection fraction | 0 (-0.009, 0.009) | | 0.99 | | 0.003 (-0.007, 0.013) | | 0.52 | | 0.003 (-0.007, 0.012) | | 0.58 | |  |
| Left atrial global peak strain | -0.022 (-0.034, -0.011) | | <0.001 | | -0.018 (-0.03, -0.006) | | **0.003** | | -0.017 (-0.028, -0.005) | | **0.007** | |  |
| **Word list sum** | | | | | | | | | | | | |  |
| E/A-ratio | 0.119 (0.031, 0.207) | | 0.008 | | 0.071 (-0.024, 0.167) | | 0.14 | | 0.07 (-0.025, 0.166) | | 0.15 | |  |
| E/e’(mean)-ratio | -0.059 (-0.149, 0.031) | | 0.20 | | 0.01 (-0.08, 0.101) | | 0.82 | | 0.011 (-0.079, 0.101) | | 0.81 | |  |
| Left atrial volume index | 0.066 (-0.034, 0.167) | | 0.19 | | 0.044 (-0.061, 0.148) | | 0.41 | | 0.042 (-0.063, 0.148) | | 0.43 | |  |
| Left atrial ejection fraction | -0.036 (-0.12, 0.047) | | 0.39 | | -0.032 (-0.119, 0.055) | | 0.47 | | -0.031 (-0.119, 0.056) | | 0.48 | |  |
| Left atrial global peak strain | 0.148 (0.06, 0.236) | | 0.001 | | 0.11 (0.02, 0.2) | | **0.017** | | 0.11 (0.019, 0.202) | | **0.019** | |  |
| **(Continued)** | | | | | | | | | | | | | |
|  | | **Model 1** | | | | **Model 2** | | | | **Model 3** | | | |
|  | | **Beta per SD increase (95% CI)** | | **p-value** | | **Beta per SD increase (95% CI)** | | **p-value** | | **Beta per SD increase (95% CI)** | | **p-value** | |
| **Word list recall** | | | | | | | | | | | | | |
| E/A-ratio | | 0.035 (-0.01, 0.08) | | 0.13 | | 0.015 (-0.033, 0.063) | | 0.54 | | 0.012 (-0.036, 0.06) | | 0.63 | |
| E/e’(mean)-ratio | | -0.037 (-0.081, 0.007) | | 0.10 | | -0.02 (-0.065, 0.025) | | 0.38 | | -0.02 (-0.064, 0.025) | | 0.38 | |
| Left atrial volume index | | -0.008 (-0.056, 0.041) | | 0.76 | | -0.015 (-0.066, 0.036) | | 0.56 | | -0.019 (-0.069, 0.032) | | 0.47 | |
| Left atrial ejection fraction | | 0.007 (-0.035, 0.049) | | 0.74 | | -0.003 (-0.047, 0.041) | | 0.90 | | -0.001 (-0.044, 0.043) | | 0.97 | |
| Left atrial global peak strain | | 0.039 (-0.006, 0.084) | | 0.09 | | 0.028 (-0.017, 0.073) | | 0.21 | | 0.026 (-0.019, 0.071) | | 0.26 | |
| Model 1: Cox regressions analyses adjusted for age and sex; Model 2: Model 1+ additional adjustment for education level and cardiovascular risk factors/diseases (arterial hypertension, diabetes mellitus, current smoking, body mass index, total cholesterol, heart rate, prior myocardial infarction, and heart failure); Model 3: Model 2 + additional adjustment for left ventricular ejection fraction and interventricular septum thickness | | | | | | | | | | | | | |
